# Pathomechanism characterization and potential therapeutics identification for SCA3 targeting neuroinflammation

**DOI:** 10.18632/aging.103700

**Published:** 2020-11-10

**Authors:** Ya-Jen Chiu, Shu-An Lin, Wan-Ling Chen, Te-Hsien Lin, Chih-Hsin Lin, Ching-Fa Yao, Wenwei Lin, Yih-Ru Wu, Kuo-Hsuan Chang, Guey-Jen Lee-Chen, Chiung-Mei Chen

**Affiliations:** 1 Department of Life Science, National Taiwan Normal University, Taipei 11677, Taiwan; 2 Department of Neurology, Chang Gung Memorial Hospital, Chang Gung University College of Medicine, Taoyuan 33302, Taiwan; 3 Department of Chemistry, National Taiwan Normal University, Taipei 11677, Taiwan

**Keywords:** spinocerebellar ataxia 3/ATXN3, IL-1β, IkBα/P65, JNK/JUN, P38/STAT1, therapeutics

## Abstract

Polyglutamine (polyQ)-mediated spinocerebellar ataxias (SCA) are caused by mutant genes with expanded CAG repeats encoding polyQ tracts. The misfolding and aggregation of polyQ proteins result in increased reactive oxygen species (ROS) and cellular toxicity. Inflammation is a common manifestation of oxidative stress and inflammatory process further reduces cellular antioxidant capacity. Increase of activated microglia in the pons of SCA type 3 (SCA3) patients suggests the involvement of neuroinflammation in the disease pathogenesis. In this study, we evaluated the anti-inflammatory potentials of indole compound NC009-1, 4-aminophenol-arachidonic acid derivative AM404, quinoline compound VB-037 and chalcone-coumarin derivative LM-031 using human HMC3 microglia and SCA3 ATXN3/Q_75_-GFP SH-SY5Y cells. The four tested compounds displayed anti-inflammatory activity by suppressing NO, IL-1β, TNF-α and IL-6 production and CD68 expression of IFN-γ-activated HMC3 microglia. In retinoic acid-differentiated ATXN3/Q_75_-GFP SH-SY5Y cells inflamed with IFN-γ-primed HMC3 conditioned medium, treatment with the tested compounds mitigated the increased caspase 1 activity and lactate dehydrogenase release, reduced polyQ aggregation and ROS and/or promoted neurite outgrowth. Examination of IL-1β- and TNF-α-mediated signaling pathways revealed that the tested compounds decreased IκBα/P65, JNK/JUN and/or P38/STAT1 signaling. The study results suggest the potential of NC009-1, AM404, VB-037 and LM-031 in treating SCA3 and probable other polyQ diseases.

## INTRODUCTION

In polyglutamine (polyQ)-mediated hereditary spinocerebellar ataxias (SCAs) types 1, 2, 3, 6, 7, 8, 17, dentatorubral-pallidoluysian atrophy (DRPLA) and Huntington’s disease (HD), abnormal expansions of the polyQ stretch in disease-causing proteins trigger misfolding of these proteins and interfere with diverse cellular processes [1, 2]. SCAs are characterized by cerebellar dysfunction alone or in combination with other neurological abnormalities [3–5]. Among SCAs, SCA3 is caused by an allele containing expanded repeats longer than 52 in the ataxin 3 (ATXN3) gene [6], a deubiquitinating enzyme that can bind and edit mixed linkage ubiquitin chains [7]. SCA3 is the most common form of SCA in Taiwan [8] and worldwide [9].

Expansion of the polyQ track in ATXN3 protein likely induces a conformational change to affect its subcellular localization and propensity to aggregate [10]. In addition, transcriptional dysregulation [11, 12], decreased anti-oxidative capacity [13–15], DNA repair dysfunction [16] and impaired ubiquitin proteasome and autophagy activity [17, 18] play important roles in pathogenesis of SCA3. The misfolded and aggregated ATXN3 protein results in a concomitant increase in reactive oxygen species (ROS) levels and cellular toxicity [13–15, 19, 20]. Inflammation is one of the manifestations of oxidative stress and inflammatory process may further induce oxidative stress and reduce cellular antioxidant capacity. In pontine neurons of SCA3 patients, expression of pro-inflammatory cytokines such as interleukin (IL)-1 receptor antagonist and IL-1β was increased, accompanied with increased numbers of reactive astrocytes and activated microglial cells [21, 22]. A reduced immune defense was also seen in phenotypic SCA3 mice [23]. Overexpression of cystathionine γ-lyase decreases oxidative stress and dampens the immune response, which could improve SCA3-associated fly eye degeneration [24]. In addition, neuropeptide Y ameliorates neuropathology and motor deficits via upregulating brain derived neurotrophic factor (BDNF) and reducing neuroinflammation markers IL-6 and induction of brown adipocytes 1 (Iba1) in SCA3 mouse models [25].

In IL-1β signaling, the inhibitor of kappa B (IκBα) protein inactivates the NF-κB transcription factor (P65/P50 heterodimer) by masking the nuclear localization signal of NF-κB and keeping it sequestered in an inactive state in the cytoplasm [26]. Specifically, IκB kinase (IKK) phosphorylates the inhibitory IκBα protein [27], resulting in the dissociation of IκBα from NF-κB. NF-κB then migrates into the nucleus and activates the expression of pro-inflammatory cytokines and chemokines, such as tumor necrosis factor (TNF)-α, IL-6 and C-C motif chemokine ligand 2 (MCP1) [28]. In addition, c-Jun N-terminal kinase (JNK)/Jun proto-oncogene, AP-1 transcription factor subunit (JUN) and mitogen-activated protein kinase 14 (P38)/signal transducer and activator of transcription 1 (STAT1) are two other transduction pathways downstream to IL-1β and TNF-α signaling, activated to up-regulate the synthesis and secretion of inflammatory factors [29, 30].

Protein aggregation, oxidative stress and neuroinflammation are common themes in neurodegenerative diseases including polyQ SCAs and Alzheimer’s disease (AD). Small heat shock proteins interact with misfolded protein aggregates, like Aβ aggregates in AD and polyQ aggregates in SCAs, to reduce the toxicity or increase the clearance of these protein aggregates [31]. To search for polyQ SCAs-modifying interventions targeting neuroinflammation, four in-house or outsourcing compounds activating molecular chaperone heat shock protein family B (small) member 1 (HSPB1) to reduce Aβ or Tau protein misfolding and aggregation were tested in this study: indole compound NC009-1 (C_19_H_16_N_2_O_3_) [32, 33], anandamide transport inhibitor AM404 (C_26_H_37_NO_2_) [34], quinoline compound VB-037 (C_24_H_20_N_4_O_3_) [35] and chalcone-coumarin derivative LM-031 (C_16_H_10_O_4_) [36, 37]. In addition, NC009-1 could reduce SCA17 polyQ aggregation by enhancing expression of HSPB1 chaperone [38]. We examined the anti-inflammatory effects of these four compounds on human HMC3 microglia [39] and SH-SY5Y cells with inducible SCA3 ATXN3/Q_75_-GFP expression, which we have established previously [40]. We also explored if these four compounds exert their effects via targeting the IL-1β- and TNF-α-mediated IκBα/P65, JNK/JUN and/or P38/STAT1 pathways.

## RESULTS

### Tested compounds, cytotoxicity, and radical scavenging activity

**Figure 1:**
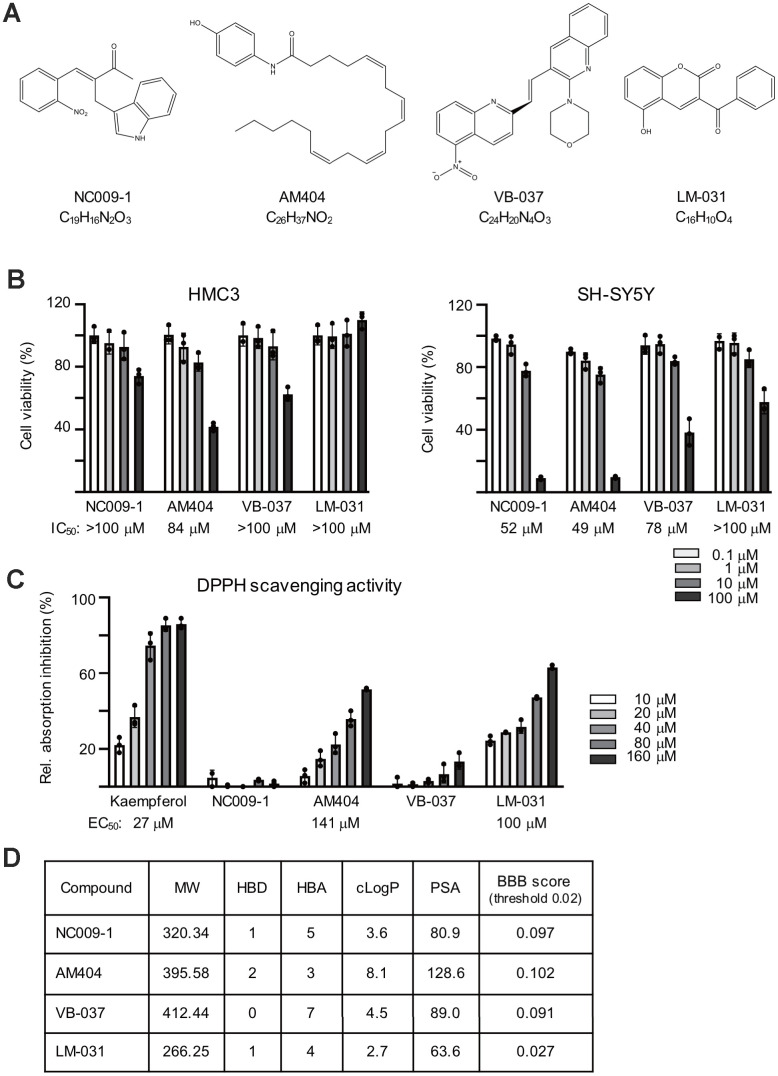
**Compounds tested.** (**A**) Structure and formula of NC009-1, AM404, VB-037 and LM-031. (**B**) Cytotoxicity of the tested compounds against HMC3 and SH-SY5Y cells using MTT viability assay. Cells were treated with 0.1−100 μM tested compounds and cell proliferation was measured after 28 h of treatment in HMC3 cells or 6 days of treatment in SH-SY5Y cells (n = 3). The IC_50_ of each compound was shown under the columns. To normalize, the relative viability in untreated cells is set as 100%. (**C**) Radical-scavenging activity of these compounds (10−160 μM) on DPPH (n = 3). (**D**) Molecular weight (MW), hydrogen bond donor (HBD), hydrogen bond acceptor (HBA), calculated octanol-water partition coefficient (cLogP), polar surface area (PSA), and predicted blood-brain barrier (BBB) score of these compounds.

Four compounds known to up-regulate HSPB1 molecular chaperone were tested (Figure 1A). Compound cytotoxicity assays were performed with human HMC3 and SH-SY5Y cells after treatment with these compounds (0.1−100 μM) for 28 h (HMC3 cells) or 6 days (SH-SY5Y cells), the treatment time for the following experiments. The calculated IC_50_ for NC009-1, AM404, VB-037 and LM-031 were >100/52, 84/49, >100/78 and >100/>100 μM, respectively, in HMC3/SH-SY5Y cells (Figure 1B). As all the tested compounds had at least 75% cell viability up to the tested 10 μM in both cells, the results demonstrated low cytotoxicity of the tested compounds on HMC3 and SH-SY5Y cells under the present experimental condition. To evaluate the radical scavenging activity of these compounds, 1,1-Diphenyl-2-picrylhydrazyl (DPPH) radical scavenging assay was conducted using kaempferol as a positive control [41]. As shown in Figure 1C, whereas no detectable DPPH-scavenging activity was seen with NC009-1 and VB-037, AM404 and LM-031 had an EC_50_ of 141 and 100 μM, respectively. Based on molecular weight (MW), hydrogen bond donors (HBD), hydrogen bond acceptors (HBA) and calculated octanol–water partition coefficient (cLogP), NC009-1, VB-037 and LM-031 meet Lipinski’s criteria in predicting a good oral bioavailability [42] (Figure 1D). With a polar surface area (PSA) less than 90 Å^2^, these three compounds were predicted to diffuse across the blood–brain barrier (BBB) [43], as also suggested by the online BBB predictor [44] (Figure 1D).

### Anti-inflammatory activity of the tested compounds on human HMC3 microglia

**Figure 2:**
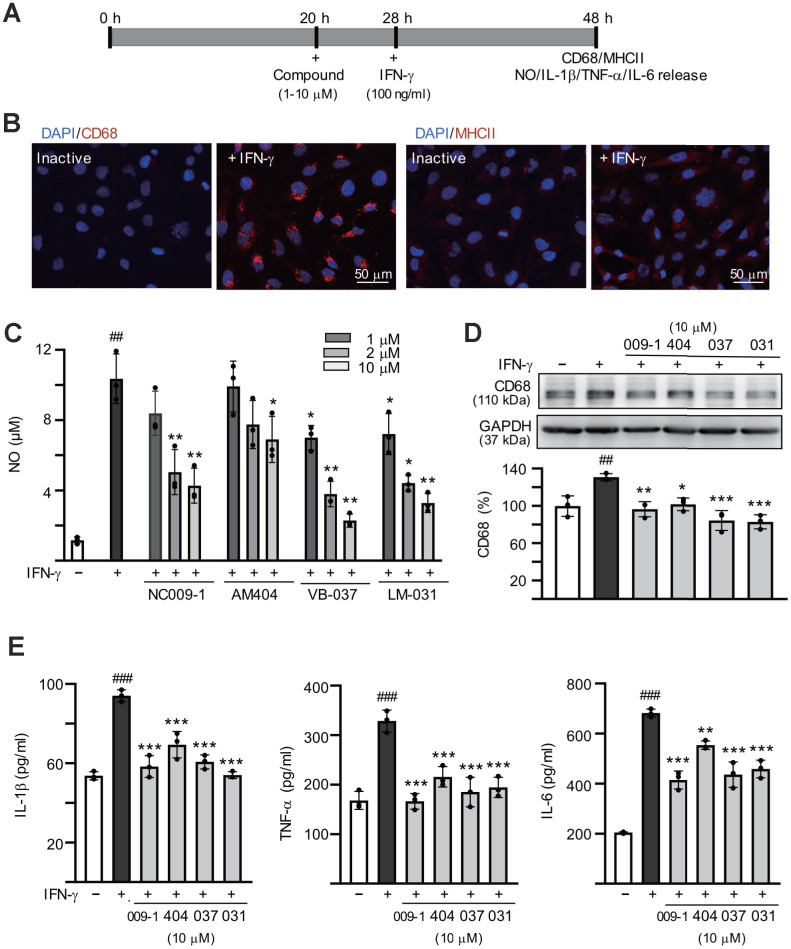
**Anti-inflammatory activities of the tested compounds on human HMC3 microglia.** (**A**) Experimental flow chart. HMC3 cells were pretreated with or without each of the tested compounds (1−10 μM) for 8 h, followed by addition of IFN-γ (100 ng/ml) to induce inflammation. After 20 h, CD68 and HMCII expression in cells as well as NO, IL-1β, TNF-α and IL-6 release in culture media were examined. (**B**) HMC3 cells with or without IFN-γ activation were analyzed by immunofluorescence using antibodies against CD68 and HMCII (red). Cell nuclei were counterstained with DAPI (blue). (**C**) Levels of NO released into culture media were measured by Griess reagent (n = 3). (**D**) Relative CD68 levels in cells treated with compound (10 μM) or not were analyzed by immunoblotting, using GAPDH as a loading control (*n* = 3). (**E**) IFN-γ-activated HMC3 cells were pretreated with the tested compounds (10 μM) and relative levels of IL-1β, TNF-α and IL-6 released into culture media were assessed by ELISA (n = 3). For normalization, the relative CD68, IL-1β, TNF-α and IL-6 levels of untreated cells (no IFN-γ activation) were set as 100%. *P* values: comparisons between IFN-γ activated and inactive cells (^##^: *P* < 0.01 and ^###^: *P* < 0.001) or between compound treated and untreated cells (*: *P* < 0.05, **: *P* < 0.01 and ***: *p* < 0.001). (one-way ANOVA with a *post hoc* Tukey test).

The anti-inflammatory responses of these compounds were examined using interferon (IFN)-γ stimulated HMC3 microglial cells [45] (Figure 2A). Exposure of HMC3 cells to IFN-γ resulted in increased expression of CD68 molecule (CD68) and major histocompatibility complex II (MHCII) (Figure 2B). The production of nitric oxide (NO) in the cultured medium was significantly increased after IFN-γ stimulation (from 1.2 μM to 10.4 μM, *P* < 0.001), whereas treatment with NC009-1 (2–10 μM), AM404 (10 μM), VB-037 (1–10 μM) and LM-031 (1–10 μM) significantly reduced NO production (from 10.4 μM to 7.2–2.3 μM; *P* = 0.043–0.005) (Figure 2C). The elevations in CD68, IL-1β, TNF-α and IL-6 were reduced significantly following treatment of these compounds at 10 μM concentration (CD68: from 131% to 102–83%, *P* = 0.011–<0.001; IL-1β: from 94 pg/ml to 69–54 pg/ml, *P* < 0.001; TNF-α: from 328 pg/ml to 215–166 pg/ml, *P* < 0.001; IL-6: from 682 pg/ml to 554–414 pg/ml, *P* = 0.003–<0.001) (Figure 2D, 2E). These results suggested that NC009-1, AM404, VB-037 and LM-031 were able to inhibit the microglial activation.

### Reduction of polyQ aggregation and promotion of neurite outgrowth of the tested compounds

ATXN3/Q_75_-GFP SH-SY5Y cells were used to examine the polyQ aggregation-inhibitory and neurite outgrowth-promoting effects of these compounds. As shown in Supplementary Figure 1, both GFP and ATXN3 antibodies detected 57 kDa ATXN3/Q_75_-GFP proteins upon doxycycline addition and the induced ATXN3/Q_75_-GFP formed aggregates in ~3% neuronal cells. For ATXN3/Q_75_-expressed cells, significantly shorter neurite total length (21.7 μm vs. 39.6 μm, *P* = 0.005) as well as less process (primary neurite, a projection from the cell body of a neuron) (1.7 vs. 2.4, *P* = 0.002) and branch (an extension from primary neurite) (0.9 vs. 2.2, *P* = 0.005) were observed compared to ATXN3/Q_14_-expressed cells. To explore the potential of NC009-1, AM404, VB-037 and LM-031 in SCA3 polyQ aggregation-inhibition and neurite outgrowth-promotion, the retinoic acid-differentiated ATXN3/Q_75_-GFP cells were treated with the tested compounds (10 μM) for 8 h and ATXN3/Q_75_-GFP expression induced (by doxycycline) for 6 days. In addition, ATXN3/Q_75_-GFP-non-expressing cells was included for comparison. Cell viability, caspase 1 activity, aggregation and neurite outgrowth were analyzed (Figure 3A). As shown in Figure 3B, neither induced ATXN3/Q_75_-GFP expression nor compound addition reduced the viability of ATXN3/Q_75_-GFP SH-SY5Y cells (99–100%, *P* > 0.05). However, induced ATXN3/Q_75_-GFP expression increased caspase 1 activity in ATXN3/Q_75_-GFP SH-SY5Y cells (128%, *P* < 0.001), whereas application of NC009-1, VB-037 or LM-031 attenuated the caspase 1 activity (114–100% vs. 128%, *P* = 0.002–<0.001). In ATXN3/Q_75_-GFP-expressing cells, treatment with NC009-1, VB-037 or LM-031 led to 12–25% reduction of aggregation (from 2.8% to 2.5–2.1%, *P* = 0.018–0.002) (Figure 3C). When the protein samples were subjected to filter trap and Western blot assays and stained with GFP antibody, reduced SDS-insoluble aggregates (58−37% vs. 100%, *P* < 0.001) and increased soluble ATXN3/Q_75_-GFP protein (144−173% vs. 100%, *P* < 0.001) were evident in samples treated with NC009-1, VB-037 or LM-031 (Figure 3C). In addition, increased neurite length (from 23.6 μm to 26.9–27.2 μm, *P* = 0.002–<0.001), process (from 1.8 to 2.0–2.1, *P* = 0.059–<0.001) and branch (from 1.0 to 1.2, *P* = 0.002–<0.001) was observed in NC009-1, VB-037 or LM-031-treated cells (Figure 3C). Although not reducing ATXN3/Q_75_ aggregation, AM404 significantly increased neurite length (30.8 μm, *P* < 0.001), process (2.2, *P* < 0.001) and branch (1.4, *P* < 0.001). In ATXN3/Q_75_-GFP-non-expressing cells, no aggregation was observed (data not shown) and neurite morphology (total length, process, and branch) was not affected by compound treatment (Figure 3D). Representative microscopy images of ATXN3/Q_75_-GFP cells induced with doxycycline, untreated or treated with the tested compounds are shown in Figure 3E and Supplementary Figure 2. These results demonstrate the aggregation-inhibitory and/or outgrowth-promoting effects of these compounds on differentiated neurons expressing ATXN3/Q_75_-GFP.

**Figure 3:**
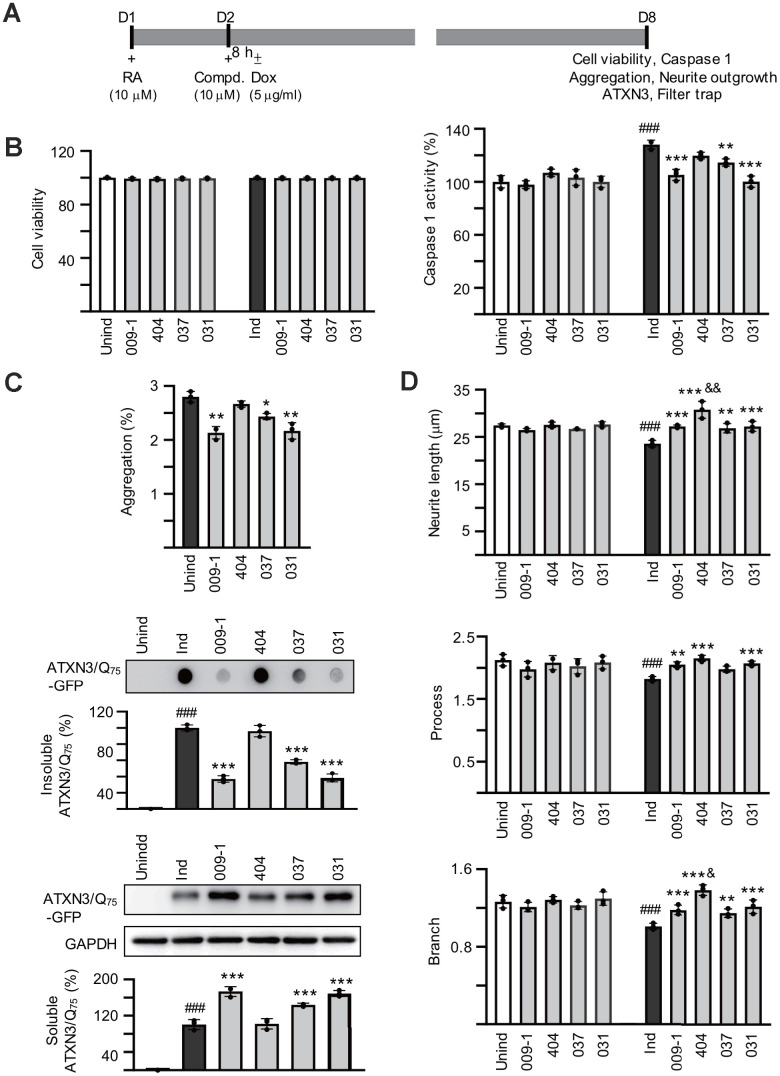
**Effects of the tested compounds on cell viability, caspase 1 activity, polyQ aggregation, and neurite outgrowth in ATXN3/Q_75_-GFP SH-SY5Y cells.** (**A**) Experimental flow chart. ATXN3/Q_75_-GFP cells were plated on dishes with retinoic acid (RA, 10 μM) added on day 1 to initiate neuronal differentiation. Next day, compound (10 μM) was added to the cells for 8 h followed by inducing ATXN3/Q_75_-GFP expression with doxycycline or not (± Dox, 5 μg/ml) for 6 days. Cell viability, caspase 1 activity, aggregation and neurite outgrowth were assessed. (**B**) Relative cell viability and caspase 1 activity (*n* = 3). For normalization, the relative viability and caspase 1 activity of uninduced and untreated cells was set as 100%. (**C**) High content polyQ aggregation analysis of ATXN3/Q_75_-GFP-expressing cells with compound treatment (*n* = 3). Shown below were filter trap assay of SDS-insoluble ATXN3/Q_75_-GFP aggregate and Western blot analysis of soluble ATXN3/Q_75_-GFP protein with compound treatment using GFP antibody (n = 3). To normalize, the relative trapped or soluble ATXN3/Q_75_-GFP without compound addition was set as 100%. (**D**) Neurite length, process or branch of ATXN3/Q_75_-GFP-non-expressing or expressing cells with compound treatment (*n* = 3).

**Figure 3:**
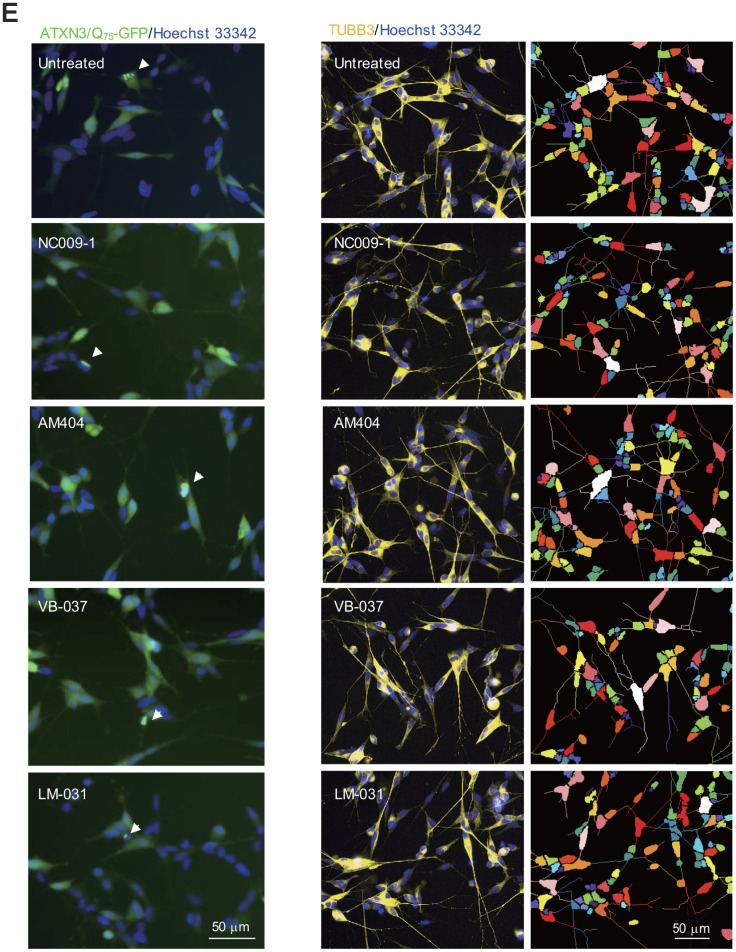
**Effects of the tested compounds on cell viability, caspase 1 activity, polyQ aggregation, and neurite outgrowth in ATXN3/Q_75_-GFP SH-SY5Y cells.** (**E**) Representative microscopic images of differentiated ATXN3/Q_75_-GFP-expressing SH-SY5Y cells, untreated or treated with NC009-1, AM404, VB-037 or LM-031, with nuclei counterstained with Hoechst 33342 (blue). Left panel: Aggregates marked with white arrowheads. Right panel: Neurite length, process and branch of TUBB3-stained ATXN3/Q_75_-GFP cells, with images segmented with multi-colored mask to assign each outgrowth to a cell body for neurite outgrowth quantification. *P* values: comparisons between untreated (induced) and uninduced cells (^###^: *P* < 0.001), or between compound treated and untreated cells (*: *P* < 0.05, **: *P* < 0.01 and ***: *P* < 0.001). (one-way ANOVA with a *post hoc* Tukey test).

### Effects of the tested compounds on conditioned medium-inflamed ATXN3/Q_75_-GFP SH-SY5Y cells

Retinoic acid-differentiated ATXN3/Q_75_-GFP SH-SY5Y cells were pretreated with the tested compounds (10 μM) for 8 h followed by ATXN3/Q_75_ induction for 6 days, and added with HMC3 conditioned medium stimulated with IFN-γ (CM/+IFN-γ) or not (CM/–IFN-γ) at a 1:1 ratio to provoke inflammatory damage to ATXN3/Q_75_-GFP-expressing SH-SY5Y cells in the last two days (Figure 4A). Figure 4B shows that CM/+IFN-γ addition reduced the viability of ATXN3/Q_75_-GFP SH-SY5Y cells (83%, *P* < 0.001) and application of these compounds rescued the decreased cell viability caused by CM/+IFN-γ addition (87–91% vs. 83%, *P* = 0.047–<0.001). Addition of CM/+IFN-γ also increased caspase 1 activity (157%, *P* < 0.001) and LDH release (146%, *P* < 0.001) of ATXN3/Q_75_-GFP SH-SY5Y cells, whereas application of these compounds attenuated the caspase 1 activity (127–90% vs. 157%, *P* = 0.045–<0.001) and LDH release (126–72% vs. 146%, *P* = 0.002–<0.001).

**Figure 4:**
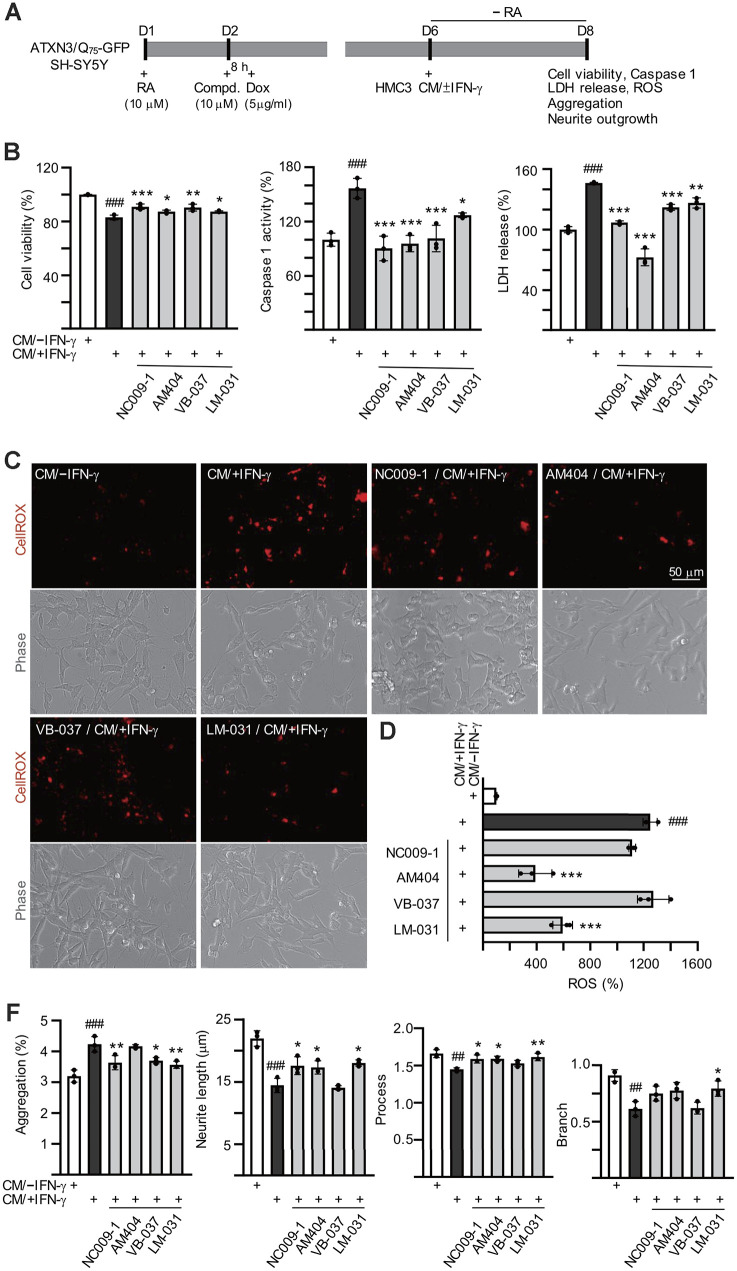
**Effects of the tested compounds in ATXN3/Q_75_-GFP-expressing SH-SY5Y cells inflamed with IFN-γ-stimulated HMC3 conditioned medium.** Experimental flowchart (**A**). ATXN3/Q_75_-GFP SH-SY5Y cells were plated in media with retinoic acid (RA, 10 μM) on day 1, and treated with the tested compound (10 μM) next day for 8 h, followed by doxycycline addition (Dox, 5 μg/ml) to induce ATXN3/Q_75_ expression. On day 6, DMEM-F12 medium without retinoic acid addition (− RA) was mixed with HMC3 conditioned medium with or without IFN-γ stimulation (CM/+IFN-γ or CM/–IFN-γ, 1:1 ratio) and added to the cells for 2 days. Cell viability, caspase 1 activity, LDH release (**B**), ROS (**D**), polyQ aggregation, neurite length, process and branch (**F**) were assessed on day 8 (n = 3). For normalization, the relative cell viability, caspase 1 activity, LDH release and ROS levels of cells treated with CM/–IFN-γ were set as 100%. (**C**) Images of ROS assay using CellROX dye (red).

**Figure 4:**
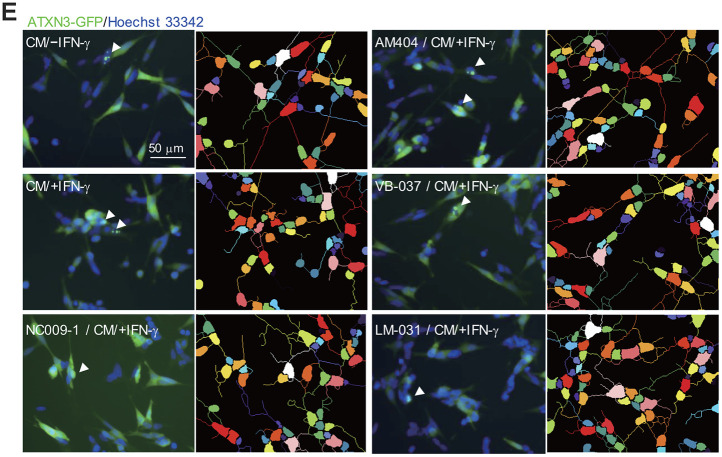
**Effects of the tested compounds in ATXN3/Q_75_-GFP-expressing SH-SY5Y cells inflamed with IFN-γ-stimulated HMC3 conditioned medium.** (**E**) Images of polyQ aggregation and neurite outgrowth, with aggregates marked with arrowheads (white), and segmented images with multi-colored mask to assign each outgrowth to a cell body for neurite outgrowth quantification. *P* values: comparisons between cells stimulated with CM/+IFN-γ and CM/–IFN-γ (^##^: *P* < 0.01 and ^###^: *P* < 0.001), or between compound treated and untreated cells (*: *P* < 0.05, **: *P* < 0.01 and ***: *P* < 0.001). (one-way ANOVA with a *post hoc* Tukey test).

ATXN3-containing polyQ expansion may increase cellular ROS levels [15]. To evaluate whether the tested compounds reduced oxidative stress in CM/+IFN-γ-inflamed ATXN3/Q_75_-GFP SH-SY5Y cells, the cellular ROS production was measured using CellROX dye. As shown in Figure 4C, 4D and Supplementary Figure 3, significantly increased ROS production (1248%, *P* < 0.001) was observed in ATXN3/Q_75_-GFP SH-SY5Y cells added with CM/+IFN-γ. Among the compounds tested, AM404 and LM-031 significantly ameliorated oxidative stress induced by CM/+IFN-γ addition (594–390% vs. 1248%, *P* < 0.001). Addition of CM/+IFN-γ significantly increased ATXN3/Q_75_ aggregation compared to the untreated cells (from 3.2% to 4.3%, *P* < 0.001) and treatment of NC009-1, VB-037 or LM-031 led to 17–22% reduction of aggregation (from 4.3% to 3.7–3.5%, *P* = 0.017–0.003) in inflamed ATXN3/Q_75_-GFP-expressing cells (Figure 4E, 4F and Supplementary Figure 4). Addition of CM/+IFN-γ also significantly reduced neurite length (from 22.0 μm to 14.4 μm, *P* < 0.001), process (from 1.7 to 1.4, *P* = 0.001) and branch (from 0.9 to 0.6, *P* = 0.001) in ATXN3/Q_75_ cells compared to the untreated cells, and treatment of NC009-1, AM404 or LM-031 increased neurite length (from 14.4 μm to 17.3–18.0 μm, *P* = 0.046–0.011) and process (from 1.4 to 1.6, *P* = 0.029–0.008), and treatment of LM-031 increased neurite branch (from 0.6 to 0.8, *P* = 0.034) (Figure 4E, 4F and Supplementary Figure 4). These results demonstrate that the tested compounds could protect cells from cell death, reduce ATXN3/Q_75_ aggregation and/or improve neurite outgrowth in inflamed ATXN3/Q_75_-GFP-expressing cells.

### Down-regulation of IL-1β- and TNF-α-mediated pathways by the tested compounds in inflamed ATXN3/Q_75_-GFP SH-SY5Y cells

**Figure 5:**
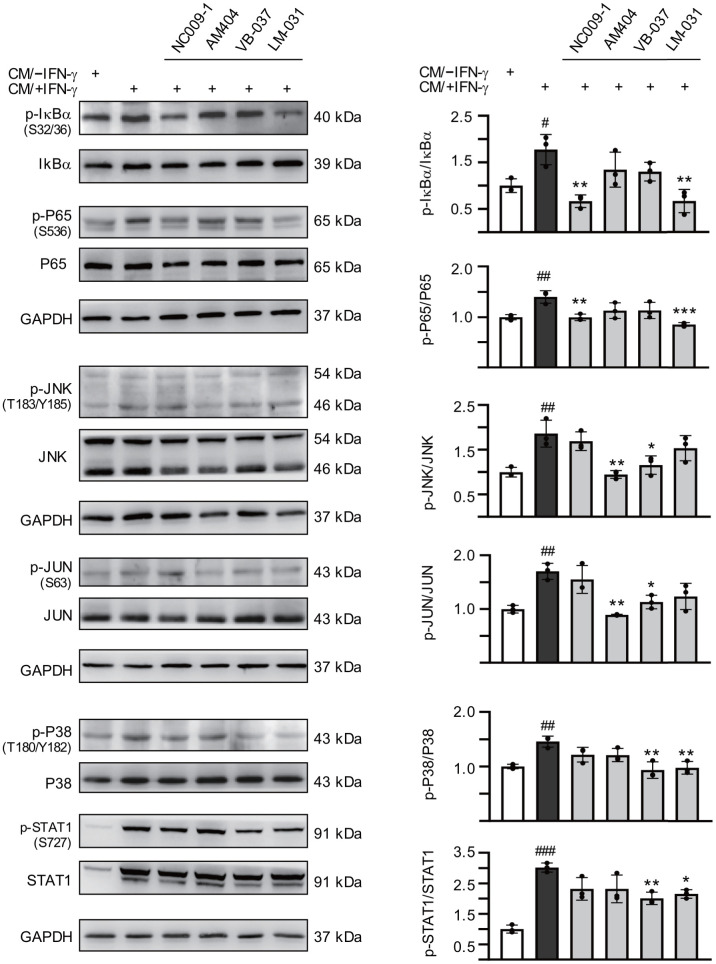
**Effects of the tested compounds on IL-1β- and TNF-α-mediated pathways in inflamed ATXN3/Q_75_-GFP SH-SY5Y cells.** Retinoic acid (10 μM)-differentiated ATXN3/Q_75_-GFP SH-SY5Y cells were treated with the tested compound (10 μM) for 8 h, followed by inducing ATXN3/Q_75_ expression for 6 days, with retinoic acid removal and HMC3 conditioned medium addition (CM/+IFN-γ or CM/–IFN-γ, 1:1 ratio) for the last 2 days. IL-1β-mediated pathways including IκBα (S32/36), P65 (S536), JNK (T183/Y185), JUN (S63), P38 (T180/Y182), and STAT1 (S727) ratios were examined (n = 3). To normalize, the relative phospho/total ratio of cells without CM stimulation was set at 100%. *P* values: comparisons between cells stimulated with CM/+IFN-γ and CM/–IFN-γ (^#^: *P* < 0.05, ^##^: *P* < 0.01 and ^###^: *P* < 0.001), or between compound treated and untreated cells (*: *P* < 0.05, **: *P* < 0.01 and ***: *P* < 0.001). (one-way ANOVA with a *post hoc* Tukey test).

Expression of pro-inflammatory IL-1β was increased in pontine neurons of SCA3 patients [21] and level of IL-1β was elevated in IFN-γ-primed HMC3 conditioned medium (Figure 2E). Upon binding to the IL-1 receptor and accessory proteins, IL-1β triggers activation of P38 and JNK mitogen-activated protein kinase (MAPK) pathways [46], both of which play a critical role in inflammatory cell signaling [47, 48]. In addition, IL-1 itself is a strong inducer of NF-κB activity [49]. Therefore, we examined the expression of these signaling pathways in CM/+IFN-γ-inflamed ATXN3/Q_75_-GFP SH-SY5Y cells by immunoblotting using specific antibodies. As shown in Figure 5, phospho/total ratios of IκBα (178%, *P* = 0.026), P65 (140%, *P* = 0.007), JNK (186%, *P* = 0.004), JUN (170%, *P* = 0.003), P38 (146%, *P* = 0.005) and STAT1 (302%, *P* < 0.001) were significantly increased after addition of CM/+IFN-γ to differentiated ATXN3/Q_75_-GFP-expressing SH-SY5Y cells, whereas treatment with NC009-1 or LM-031 reduced the phospho/total ratios of IκBα (from 178% to 67–66%, *P* = 0.002) and P65 (from 140% to 99–85%, *P* = 0.006–<0.001), treatment with AM404 or VB-037 reduced the phospho/total ratios of JNK (from 186% to 116–95%, *P* = 0.016–0.002) and JUN (from 170% to 113–89%, *P* = 0.014–0.001), and treatment with VB-037 or LM-031 reduced the phospho/total ratios of P38 (from 146% to 98–93%, *P* = 0.003–0.002) and STAT1 (from 302% to 215–201%, *P* = 0.020–0.007). These results demonstrated the anti-inflammatory effects of the tested compounds on SCA3 neuronal cells.

## DISCUSSION

Several lines of evidence have shown that increased oxidative stress and decreased anti-oxidative response play a crucial role in the pathogenesis of SCA3 [13–15, 19, 20]. Increased pro-inflammatory cytokines, reactive astrocytes and activated microglia have been found in pons of SCA3 patients and mice [21, 22, 25]. Aberrant immune responses were also demonstrated in SCA3 mice and fly [23, 24]. Inflammation and microglial activation have been shown to contribute to neurotoxicity in HD [50–53], another polyQ-mediated disease. Substantial evidence has also shown that oxidative stress and inflammation interplay to confer detrimental effects on neurons [54]. Therefore, agents targeting both oxidative and inflammatory pathways may serve as a good candidate for treating diseases such as polyQ diseases including SCA3, where both inflammation and increased oxidative stress play a pivotal role in pathogenesis.

In this study, we showed anti-inflammatory, anti-oxidative and/or neuroprotective effects of NC009-1, AM404, VB-037 and LM-031. The anti-inflammatory effects of the tested compounds were demonstrated by using IFN-γ-stimulated human HMC3 microglia, where these compounds significantly decreased release of pro-inflammatory cytokines, IL-1β, TNF-α and IL-6. We then applied HMC3 conditioned medium to ATXN3/Q_75_-GFP SH-SY5Y cells to provoke inflammation-induced damaging effects including reduced cell viability and neurite outgrowth, and increased aggregation, caspase1 activity and oxidative stress, as evidently shown in Figure 4. It may be better to show if the tested compounds have effect on activation of HMC3 cells expressing ATXN3. However, this study is mainly focusing on if exogenous inflammatory stimuli exaggerate the damage of expanded polyQ on neurons and if the tested compounds rescue the cytotoxicity of the cytokines released from IFN-γ-activated HMC3 cells. Our results are in accordance with that addition of TNF-α and IL-1β induced toxicity and apoptosis of primary cortical neurons from a HD mouse model and neurons derived from HD induced pluripotent stem cells [52]. The neuroprotection effects were further shown in ATXN3/Q_75_-GFP SH-SY5Y cells inflamed by addition of CM/+IFN-γ, where NC009-1, AM404, VB-037 and LM-031 significantly increased cell viability and decreased caspase 1 activity, NC009-1, AM404 and LM-031 reduced oxidative stress and rescued the deficits of neurite outgrowth, while NC009-1, VB-037 and LM-031 ameliorated aggregation. It is noted that AM404 had significant effects on neurite length/process/branch, caspase 1 activity/LDH release and ROS level, whereas it did not reduce aggregation. The similar effects have been shown in previous studies in polyQ diseases, where treatments or compounds had significant neuroprotection, but did not effectively inhibit aggregation, which suggests the protection is acting on downstream pathological processes secondary to toxic fragments of polyQ-containing protein rather than on aggregate-inhibition [55]. Furthermore, several studies have suggested that it is the soluble fragmented protein containing expanded polyQ that is toxic rather than aggregates and aggregate-reduction alone does not necessarily rescue neurotoxicity [56–58].

It has been shown that IL-1β and TNF-α induce Purkinje neuronal apoptosis treated with conditioned medium derived from hypoxic microglia, indicating the high vulnerability of Purkinje neurons to cytokines released from microglia [59]. IL-1β- and TNF-α-mediated transduction pathways include NF-κB (P65/P50 heterodimer), JNK/JUN and P38/STAT1 signaling [46, 60]. The degradation of IκB by IKK permits translocation of NF-κB into nucleus with subsequent transcription of the downstream pro-inflammatory genes, which has a central role in immune response and inflammation-associated diseases [61]. JNK and P38, stress-activated MAPK, play an important role in inflammation-responses including further inflammatory genes transcription and cytokine production [62, 63]. Activated JNK or P38 also translocates to the nucleus to phosphorylate transcription factors such as JUN, FOS, STAT1 and MYC, which up-regulate the expression of downstream pro-apoptotic genes [62–64]. Since our study showed increased IL-1β, TNF-α and IL-6 in the medium of IFN-γ-stimulated HMC3 microglia and IL-1β has been known to promote IL-6 secretion, we then went on to examine if IL-1β- and TNF-α-mediated pathways are involved in the neurotoxicity in ATXN3/Q_75_-GFP SH-SY5Y cells inflamed by HMC3 conditioned medium. It is evident that HMC3 conditioned medium significantly increased ratios of p-IκBα/IκBα, p-P65/P65, p-JNK/JNK, p-JUN/JUN, p-P38/P38 and p-STAT1/STAT1 in ATXN3/Q_75_-GFP SH-SY5Y cells. Although inflammation such as increased IL-1β and IL-6 has been shown in the brains of SCA3 and mice, whether their downstream signaling pathways are involved is not clear [21, 22, 25], we here provide evidence that NF-κB, JNK/JUN and P38/STAT1 are activated in inflamed SCA3 cells. Treatment with NC009-1 or LM-031 significantly decreased ratios of p-IκBα/IκBα and p-P65/P65, and AM404 or VB-037 significantly reduced ratios of p-JNK/JNK and p-JUN/JUN, whereas VB-037 or LM-031 significantly ameliorated the elevated ratios of p-P38/P38 and p-STAT1/STAT1, indicating their anti-inflammatory effects on inflamed ATXN3/Q_75_-GFP SH-SY5Y cells via targeting IL-1β- and TNF-α-mediated pathways.

Treatments targeting inflammation for SCA3 have been rarely reported, although several studies have shown anti-inflammatory strategies are beneficial to other polyQ diseases [52, 65–67]. Hsiao and colleagues showed that inhibition of TNF-α improved motor function, reduced caspase activation, diminished the aggregates, increased neuronal density and decreased gliosis in the brains of R6/2 HD mice [52]. Aikawa and colleagues demonstrated that genetic ablation of myeloid differentiation factor 88 (Myd88), a major adaptor molecule essential for Toll-like receptor (TLR) signaling, ameliorated Purkinje cell loss and partially rescued motor impairments in a SCA6 mouse model [65]. Yang and colleagues also found that the NF-κB was activated in SCA17 knock–in mice and blocking NF-κB signaling in astrocytes ameliorated neurodegeneration [66]. Recently, Dubey and Tapadia have found that expanded polyQ triggered antimicrobial peptides (AMPs) expression and JNK activation, whereas Yorkie, the co-activator of the Hippo pathway, down-regulated AMPs and p-JNK to rescue apoptosis and mitigated polyQ-mediated toxicity in the eye of polyQ-expressing fly [67]. These studies suggest that enhanced inflammatory response contributes to polyQ mediated neurodegeneration and agents targeting inflammation may serve as potential therapeutics for polyQ diseases.

ROS have been reported to activate ‎extracellular signal-regulated kinases (ERKs), JNKs and P38, but the mechanisms by which ROS can activate these kinases are unclear [68]. Our study results also show that oxidative stress is increased and JNK and P38 are activated in inflamed neurons expressing expanded polyQ, supporting the proposal that ROS may also contribute to IL-1β- and TNF-α-mediated inflammation. The treatments of test compounds decrease inflammation and/or reduce oxidative stress, both of which may contribute the rescue of neurodegeneration.

Our study results demonstrated low cytotoxicity and high predicted BBB scores of all the tested compounds, suggesting their potential of serving as a treatment for neurodegenerative diseases including SCA3. Among them, NC009-1 [33, 38], AM404 [69] and LM-031 [37] have been tested for *in vivo* usages. The known mechanisms of action of these compounds in neuroprotection are summarized below. In addition to up-regulating HSPB1 chaperone, NC009-1, VB-037 and LM-031 also displayed chemical chaperone-like activity in thioflavin T assay of Aβ aggregation [35, 36, 70]. NC009-1 has been shown previously to have aggregation-reducing and neuroprotection effects by activating HSPB1 to increase pro-aggregated ΔK280 Tau_RD_ solubility and promote neurite outgrowth in tauopathy cell model [32]. Also by increasing HSPB1 expression, NC009-1 mitigated the increased BH3 interacting domain death agonist (BID), cytochrome c release, and caspase 3 activation to reduce polyQ aggregation and apoptosis in SCA17 TBP/Q_79_ cells, as well as ameliorated behavioral deficits in SCA17 TBP/Q_109_ transgenic mice [38]. Moreover, NC009-1 up-regulated apolipoprotein E (APOE) and tropomyosin receptor kinase A (TRKA) expression to improve neurite outgrowth in Aβ-GFP SH-SY5Y cell, as well as to reduce hippocampal/cortical Aβ and Tau levels and ameliorate cognitive deficits in hyperglycemic APP_Swe_/PS1_M146V_/Tau_P301L_ triple transgenic mice [33]. Here, we for the first time show the anti-inflammatory effect of NC009-1 to provide neuroprotection. AM404, an anandamide transport inhibitor, was previously selected through virtual screening compound databases to search for compounds which act as a glycogen synthase kinase-3β (GSK-3β) kinase inhibitor [34]. Through increasing phospho-GSK-3β (Ser9) expression to reduce Tau phosphorylation, AM404 enhanced HSPB1 and GRP78 (glucose-regulated protein, 78 kDa) expression, increased pro-aggregated Tau solubility, and promoted neurite outgrowth in ΔK280 Tau_RD_ AD cell model [34]. Through increasing the phosphorylation of AKT (AKT serine/threonine kinase 1) and GSK-3β, AM404 at low dose ameliorated cognitive deficit and reduced Aβ, Tau hyperphosphorylation, and inflammation in hyperglycemic 3×Tg-AD mice [69]. AM404 also inhibits directly Ca^2+^ flux of L-type voltage-dependent Ca^2+^ channels [71]. AM404 can reduce allodynia in a neuropathic pain model via cannabinoid CB1 receptor activation [72]. Attenuation of lipopolysaccharide (LPS)-induced increases in IL-1β and IL-6 by AM404, mediated through the cannabinoid CB1 receptor, has also been demonstrated [73]. Our results further support its anti-inflammatory effect, but in a SCA3 model. Recently our group has shown the anti-aggregation, anti-oxidative, and neuroprotective effects of LM-031, a novel derivative of chalcone-coumarin, against Aβ or Tau toxicity through activation of the HSPB1 chaperone, NRF2 (nuclear factor, erythroid 2 like 2)/NQO1 (NAD(P)H quinone dehydrogenase 1)/GCLC (glutamate-cysteine ligase catalytic subunit) pathway, and CREB (cAMP-response element binding protein 1)/BDNF (brain derived neurotrophic factor)/BCL2 (BCL2 apoptosis regulator) pathway [36, 37]. However, the anti-inflammatory effect of LM-031 is for the first time demonstrated in the present study. We have also previously shown that VB-037, a novel quinoline compound, attenuated LPS/IFN-γ-induced activation of BV-2 microglia and diminished LPS/IFN-γ-induced increase of caspase 1 activity, expression of IL-1β, and phosphorylation of P38, JNK and JUN to protect Aβ-GFP-expressing SHSY5Y cells against inflammatory damage [35]. Here, we again show its protection effect via anti-inflammatory action on another neurodegenerative disease model. However, it should be addressed that future studies in SCA3 animal models are warranted to further consolidate the neuroprotection effects of the tested compounds.

## CONCLUSION

**Figure 6:**
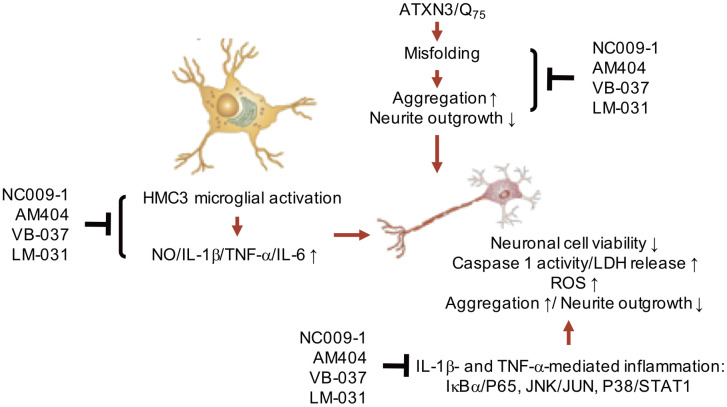
**Graphic summary.**

In summary, our study shows that neuroinflammation contributes to increased aggregation and neurotoxicity in ATXN3/Q_75_-GFP SH-SY5Y cells. NC009-1, AM404, VB-037 and LM-031 reduce aggregation, neuroinflammation and ROS, and/or promote neurite outgrowth by down-regulating IL-1β and TNF-α-mediated pathways and their downstream IκBα/P65, JNK/JUN or P38/STAT1 signaling (Figure 6). The study results shed light on the involvement of neuroinflammation in SCA3 and the potential of NC009-1, AM404, VB-037 and LM-031 in treating SCA3 and probable other polyQ diseases.

## MATERIALS AND METHODS

### Compounds and cell culture

Anandamide transport inhibitor AM404 and quinoline compound VB-037 were purchased from Sigma-Aldrich (St. Louis, MO, USA) and Enamine (Kiev, Ukraine), respectively. Indole compound NC009-1 and chalcone-coumarin derivative LM-031 were synthesized and characterized by NMR spectrum as described [36, 74]. No geometric isomers occur in both compounds. Human HMC3 microglial cells (ATCC CRL-3304) and SH-SY5Y cells (ATCC CRL-2266) were maintained in Dulbecco’s modified Eagle medium/Nutrient mixture F-12 (DMEM/F-12) supplemented with 10% fetal bovine serum (FBS) (Thermo Fisher Scientific, Waltham, MA, USA). ATXN3/Q_75_-GFP SH-SY5Y cells [40] were cultivated in DMEM/F-12 containing 10% FBS, with 5 μg/ml blasticidin and 100 μg/ml hygromycin (InvivoGen, San Diego, CA, USA) added to the growth medium.

### Cell proliferation assay

To evaluate compound cytotoxicity, 2 × 10^4^ (HMC3) or 1 × 10^4^ (SH-SY5Y) cells were plated on 48-well dishes, grown for 20 h, and treated with the tested compounds (0.1−100 μM). After 28 h (HMC3) or 6 days (SH-SY5Y), 20 μl of 3-(4,5-dimethylthiazol-2-yl)-2,5-diphenyltetrazolium bromide (MTT) (5 mg/ml) was added to the cells at 37°C for 3 h. 200 μl of lysis buffer (10% Triton X-100, 0.1 N HCl, 18% isopropanol) was then added to dishes and the absorbance of the insoluble purple formazan product at OD 570 nm was read by a FLx800 fluorescence microplate spectrophotometer (Bio-Tek, Winooski, VT, USA). The IC_50_ of the tested compounds were calculated using the interpolation method.

### Radical-scavenging assay

The DPPH radical-scavenging activity was measured in an ethanol mixture containing 200 μM DPPH (Sigma-Aldrich) radical solution and the tested compounds (10–160 μM). The solution was vortexed and incubated for 30 min at room temperature. The scavenging capacity was measured by monitoring the decrease in absorbance at 517 nm with a microplate spectrophotometer (Multiskan GO, Thermo Fisher Scientific). The half maximal effective concentrations (EC_50_) for inhibition of the formation of DPPH radicals were calculated using the interpolation method.

### Bioavailability and BBB permeation prediction

Internet software ChemDraw (http://www.perkinelmer.com/tw/category/chemdraw) was used to calculate molecular weight (MW), hydrogen bond donor (HBD), hydrogen bond acceptor (HBA), octanol-water partition coefficient (cLogP) and polar surface area (PSA). In addition, blood-brain barrier (BBB) prediction server (Online BBB Predictor, https://www.cbligand.org/BBB/) was used to calculate BBB permeation score.

### Immunocytochemical staining of CD68 and MHCII

HMC3 cells were plated into 6-well (2 × 10^5^/well) dishes containing coverslips, grown for 20 h and stimulated with IFN-γ (100 ng/ml) for 20 h. Then the cells were fixed with 4% paraformaldehyde for 30 min, permeabilized with 0.1% Triton X-100 for 10 min, and blocked non-specific binding with 3% BSA for 20 min. The primary anti-CD68 (1:1000; Cell Signaling, Danvers, MA, USA) or anti-MHCII (1:1000; Invitrogen, Waltham, MA, USA) antibody was used to stain cells at 4°C overnight, followed by Alexa Fluor 555-donkey anti-rabbit or Cy^TM^5-goat anti-mouse (IgG) secondary antibody (1:1000; Invitrogen) staining for 2 h at room temperature. Nuclei were detected using 4’,6-diamidino-2-phenylindole (DAPI; 0.1 μg/ml; Sigma-Aldrich). The stained cells were examined using Zeiss LSM 880 confocal laser scanning microscope (Zeiss, Oberkochen, Germany).

### Detection of inflammatory mediators

HMC3 cells were plated into 6-well (2 × 10^5^/well) dishes, grown for 20 h and treated with the tested compounds (1–10 μM) for 8 h followed by IFN-γ (100 ng/ml) stimulation. The release of NO in cell culture medium was evaluated by Griess assay according to manufacturer’s protocol (Thermo Fisher Scientific). In addition, the levels of IL-1β, TNF-α and IL-6 in medium pre-treated with 10 μM compound were determined using Human Instant ELISA^TM^ Kit following the manufacturer’s protocol (Invitrogen). The optical density at 450 nm was detected using Multiskan GO spectrophotometer.

### High content ATXN3 polyQ aggregation and neurite outgrowth analyses

ATXN3/Q_75_-GFP SH-SY5Y cells were plated on 24-well (1 × 10^4^/well) dishes, and retinoic acid (10 μM; Sigma-Aldrich) was added to initiate neuronal differentiation. On the second day, cells were treated with the tested compound (10 μM) for 8 h before ATXN3-GFP expression induction by adding doxycycline (5 μg/ml). For comparison, cells without inducing ATXN3/Q_75_-GFP expression were included. The cells were kept in the medium containing retinoic acid, doxycycline and test compound for 6 days. For the cells with inflammatory stimulation, HMC3 conditioned medium with (CM/+IFN-γ) or without (CM/–IFN-γ) IFN-γ stimulation was added at a 1:1 ratio in the last two days. On the eighth day, cells were stained with Hoechst 33342 (0.1 μg/ml; Sigma-Aldrich) for 30 min, and images of the cells were automatically obtained using an ImageXpressMICRO high content analysis (HCA) system (Molecular Devices, San Jose, CA, USA). Excitation/emission filters were at 482/536 and 377/447 nm for enhanced GFP and Hoechst 33342, respectively. Aggregation was determined by Transfluor technology [75] based on GFP fluorescence intensity. Neurite length, process and branch of ATXN3/Q_75_-GFP-expressing cells were analyzed by using Metamorph microscopy automation and image analysis software (neurite outgrowth application module, Molecular Devices). In addition, cells were fixed, permeated and stained with neuronal class III β-tubulin (TUBB3) antibody (1:1000; Covance, Princeton, NJ, USA), followed by anti-rabbit Alexa Fluor ^®^555 antibody (1:1000; Thermo Fisher Scientific) for neurite outgrowth analysis. To quantify neurite outgrowth, microscopic images were segmented with multi-colored mask to assign each outgrowth to a cell body for quantification. In general, 10^4^ cells in each biological replicate were analyzed.

### Cell viability, caspase 1 activity, and LDH release assays

Cell viability was assayed by propidium iodide (PI) staining. Briefly, retinoic acid-differentiated ATXN3/Q_75_-GFP SH-SY5Y cells (2 × 10^5^ on 6-well dishes) were pretreated with the tested compounds, induced ATXN3/Q_75_ expression, and inflamed with HMC3 conditioned medium (CM/+IFN-γ or CM/–IFN-γ) as described. On the eighth day, the cells were stained with PI (0.6 μg/ml; Sigma-Aldrich) and Hoechst 33342 (0.1 μg/ml) for 30 min, and images of the cells were automatically obtained using the HCA, with 535 nm excitation and 617 nm emission filters for PI. For caspase 1 activity assay, the cells were lysed by repeated freeze-thaw and supernatants collected after centrifugation at 12,000 × g for 10 min. Caspase 1 activity was measured with the caspase 1 assay kit based on the cleavage of substrate YVAD-AFC according to the manufacturer’s instructions (BioVision, Milpitas, CA, USA). The absorbance was read using FLx800 microplate reader with excitation at 400 nm and emission at 505 nm. For LDH release assay, cell culture media were collected on day 8 and the release of LDH was examined by using LDH cytotoxicity assay kit (Cayman, Ann Arbor, MI, USA). The absorbance was read at 490 nm with Multiskan GO microplate reader.

### ROS analysis

Retinoic acid-differentiated ATXN3/Q_75_-GFP SH-SY5Y cells (8 × 10^3^ on 96-well dishes) were pretreated with the tested compounds, induced ATXN3/Q_75_ expression, and inflamed with HMC3 conditioned medium as described. On the eighth day, fluorogenic CellROX deep red reagent (5 μM; Invitrogen) and Hoechst 33342 (0.1 μg/ml) were added to the cells and incubated at 37°C for 30 min. Images of the cells were obtained and analyzed using the HCA, with 640 nm excitation and 665 nm emission filters for CellROX deep red reagent.

### Western blot analysis and filter trap assay

Cells were lysed using buffer (50 mM Tris-HCl pH8.0, 0.5% sodium deoxycholate, 0.1% SDS, 150 mM NaCl, 2 mM EDTA, 50 mM NaF and 1% NP40) containing the protease inhibitor cocktail (Sigma-Aldrich). After sonication, the lysates were centrifuged at 12,000 × g for 10 min at 4°C. Protein concentrations were determined using a protein assay kit (Bio-Rad, Hercules, CA, USA), with albumin as standards. Total soluble proteins (20 μg) were electrophoresed on 10% SDS-polyacrylamide gel and transferred onto polyvinylidene difluoride membrane (Sigma-Aldrich) by reverse electrophoresis. After being blocked, the membrane was stained with ATXN3 (1:1000; GeneTex, Irvive, CA, USA), GFP (1:1000; Santa Cruz Biotechnology, Santa Cruz, CA, USA), CD68 (1:1000; Cell Signaling), IκBα (1:1000; Cell Signaling), p-IκBα (S32/36) (1:1000; Cell Signaling), P65 (1:1000; Cell Signaling), p-P65 (S536) (1:1000; Cell Signaling), JNK (1:1000; Cell Signaling), p-JNK (T183/Y185) (1:500; Cell Signaling), JUN, p-JUN (S63) (1:1000; Cell Signaling), P38 (1:1000; Cell Signaling), p-P38 (T180/Y182) (1:1000; Cell Signaling), STAT1 (1:1000; Cell Signaling), p-STAT1 (S727) (1:1000; Cell Signaling), or GAPDH (1:5000; MDBio Inc., Taipei, Taiwan) primary antibody at room temperature 2 h or 4°C overnight. The immune complexes were detected using horseradish peroxidase (HRP)-conjugated goat anti-mouse or goat anti-rabbit IgG antibody (1:5000; GeneTex) and chemiluminescent HRP substrate (Millipore, Billerica, MA, USA).

For filter trap assay, protein (20 μg) was diluted in 2% SDS in PBS and filtered through a cellulose acetate membrane (0.2 μm pore size; Merck, Kenilworth, NJ, USA) pre-equilibrated in 2% SDS in PBS on a dot-blot filtration unit (Bio-Rad Laboratories, Hercules, CA, USA). After washing with 2% SDS buffer and blocking in PBS containing 5% nonfat dried milk, the cellulose acetate membrane was probed with anti-GFP antibody (1:1000; Santa Cruz Biotechnology) and the immune complexes on the membrane were detected as described.

### Statistical analysis

For each data set, three independent experiments were performed and data were expressed as the means ± standard deviation (SD). Differences between groups were evaluated by Student’s *t* test (comparing two groups) or one-way analysis of variance with a *post hoc* Tukey test where appropriate (comparing several groups). All *P* values were two-tailed, with values lower than 0.05 to be considered statistically significant.

## Supplementary Material

Supplementary Figures
