# Supplementary material for: Pathomechanism characterization and potential therapeutics identification for SCA3 targeting neuroinflammation
Source: Aging (Albany NY). 2020 Nov 10;12(23):23619–46. doi: 10.18632/aging.103700 (PMC7762503; doi:10.18632/aging.103700)
Supplement: Supplementary Figures [file aging-12-103700-s001.pdf]

SUPPLEMENTARY FIGURES

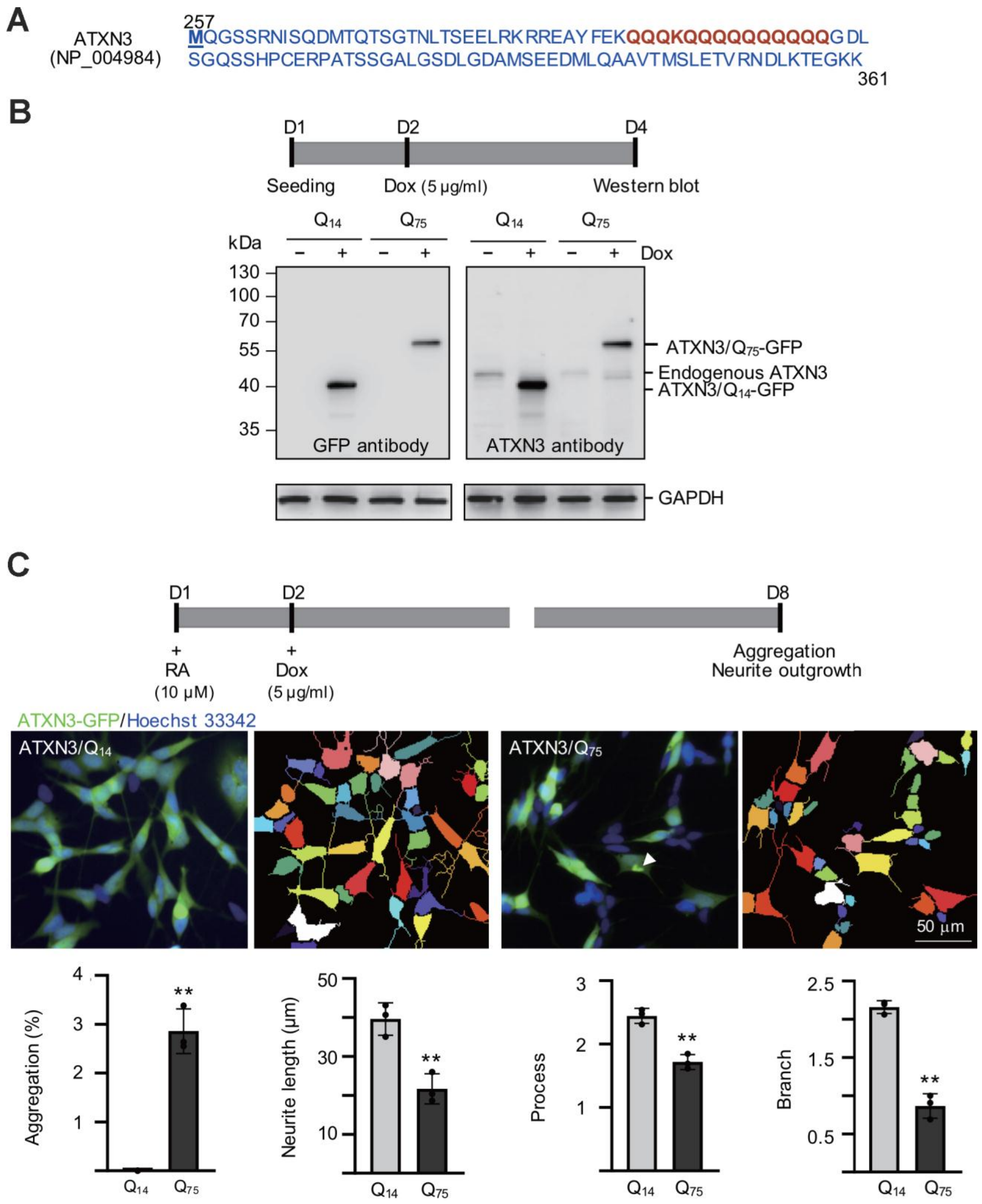

Supplementary Figure 1. ATXN3/Q_{14-75}-GFP SH-SY5Y cells. (**A**) Amino acid sequences of ATXN3/Q_{14} (residues 257–361, NP_004984). (**B**) Western blot analysis of ATXN3/Q_{14-75}-GFP SH-SY5Y cells using GFP and ATXN3 antibodies after two days of induction (+ Dox). GAPDH was included as a loading control. (**C**) Experimental flow chart of retinoic acid (RA, 10 µM) induced neuronal differentiation and microscopic images of differentiated SH-SY5Y cells expressing ATXN3/Q_{14-75}-GFP for 6 days, with relative aggregation, neurite length, process, and branch shown below. Shown next to the microscopic images are segmented images with multi-colored mask to assign each outgrowth to a cell body for neurite outgrowth quantification. *P* values: comparisons between ATXN3/Q_{14} and ATXN3/Q_{75} cells (*: $P < 0.05$, **: $P < 0.01$, and ***: $P < 0.001$). (two-tailed Student's *t* test).

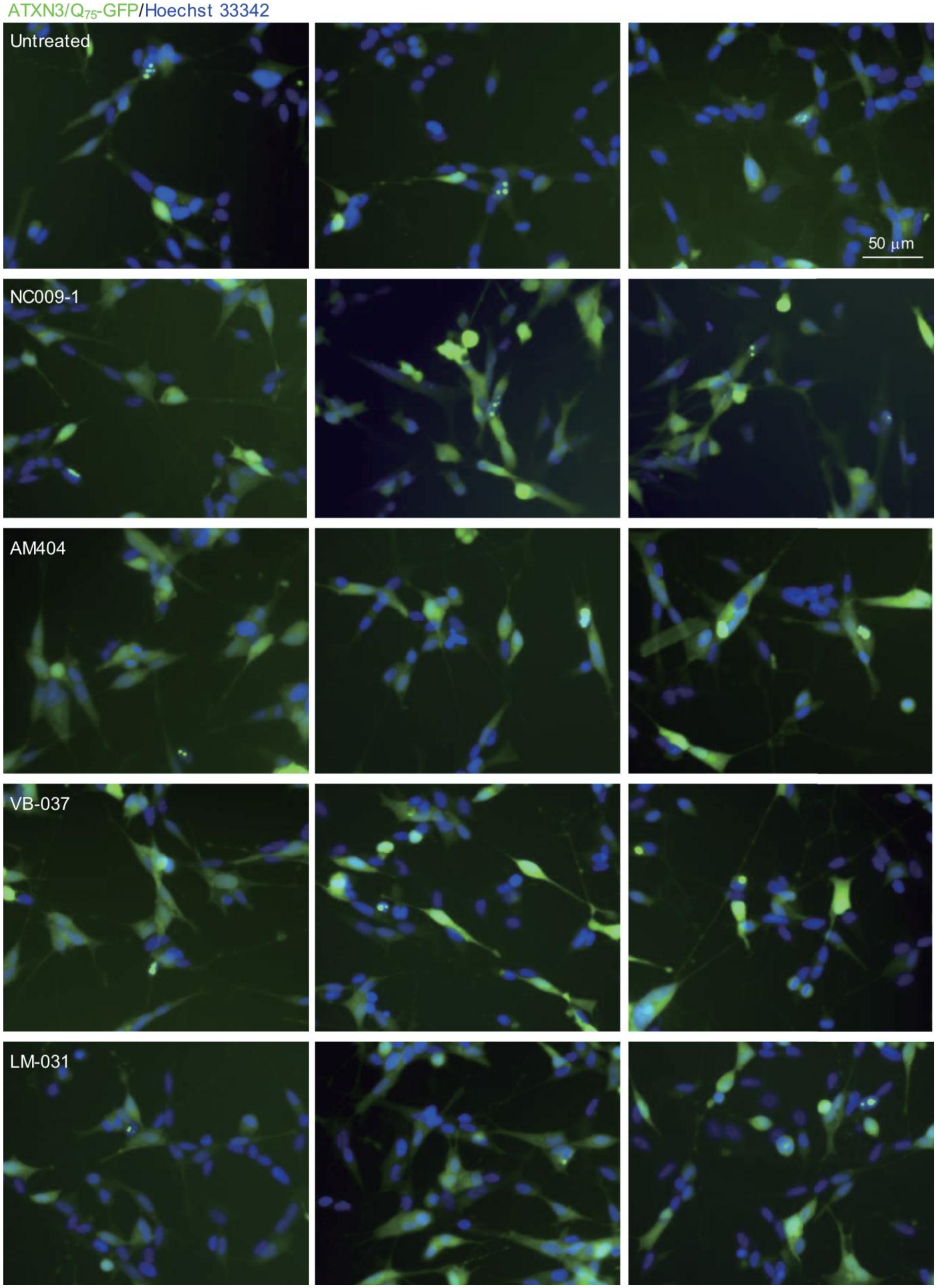
ATXN3/Q_{75}-GFP/Hoechst 33342
Untreated
50 μm
NC009-1
AM404
VB-037
LM-031

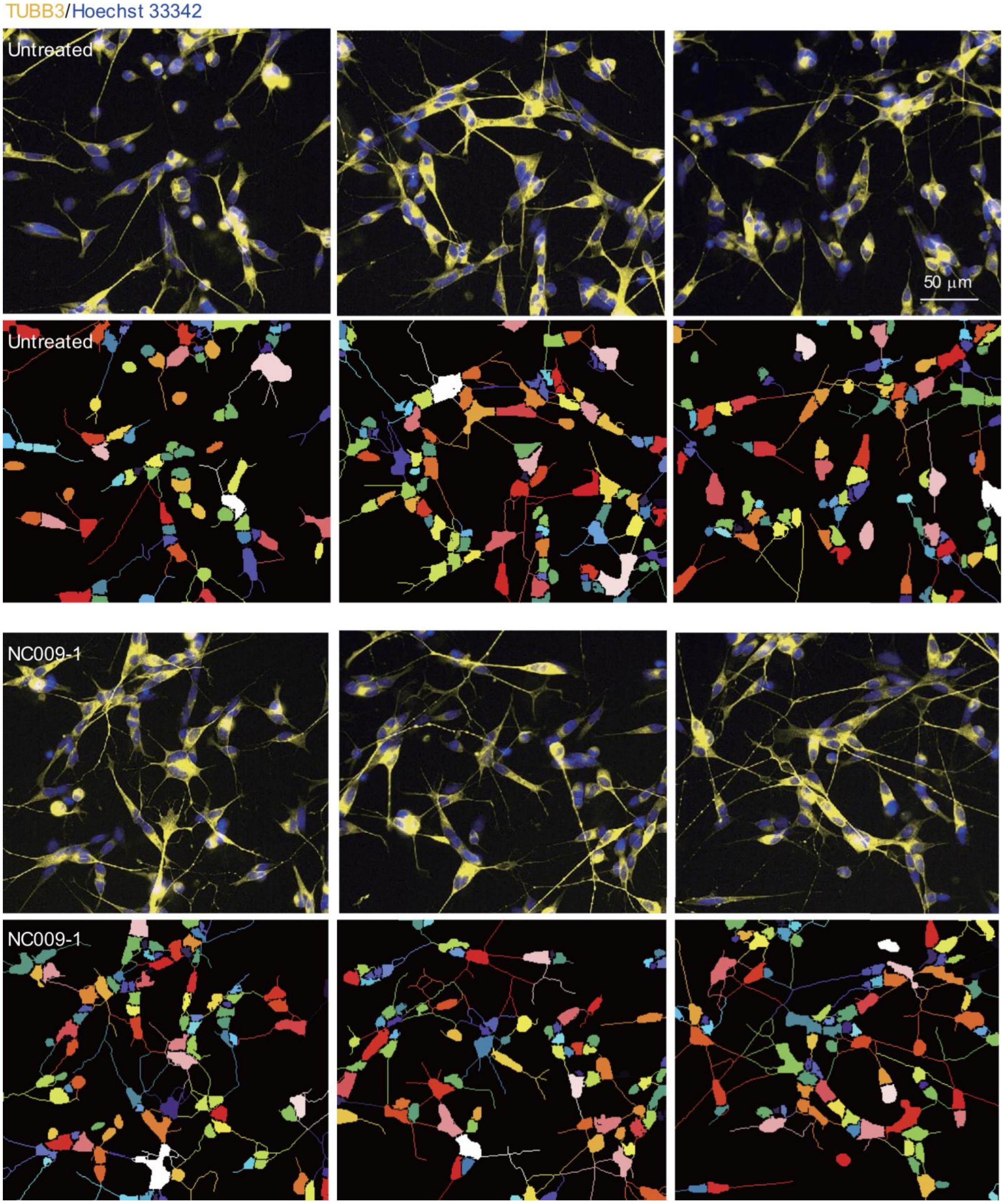
TUBB3/Hoechst 33342
Untreated
50 µm
Untreated
NC009-1
NC009-1

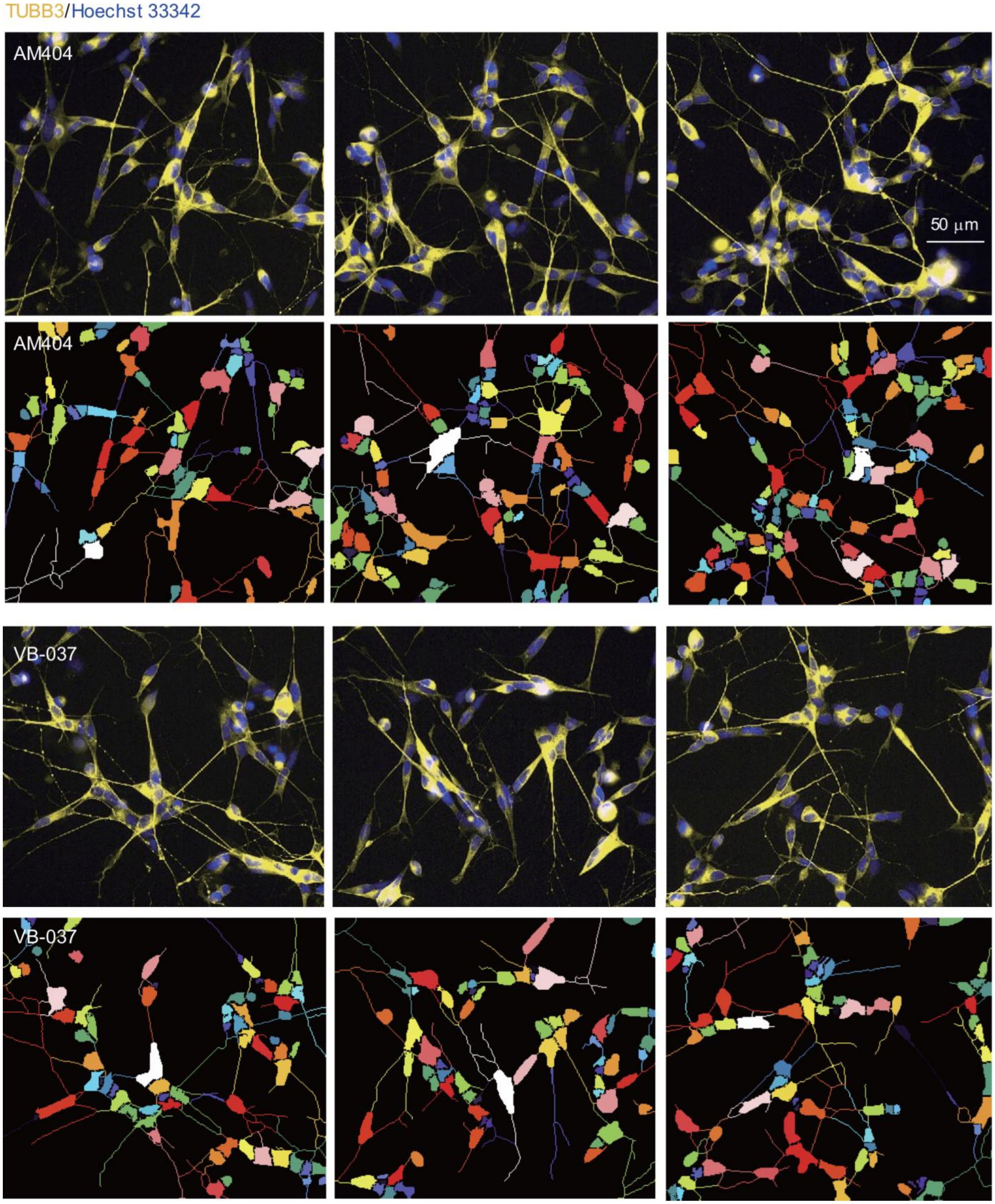
TUBB3/Hoechst 33342
AM404
50 µm
AM404
VB-037
VB-037

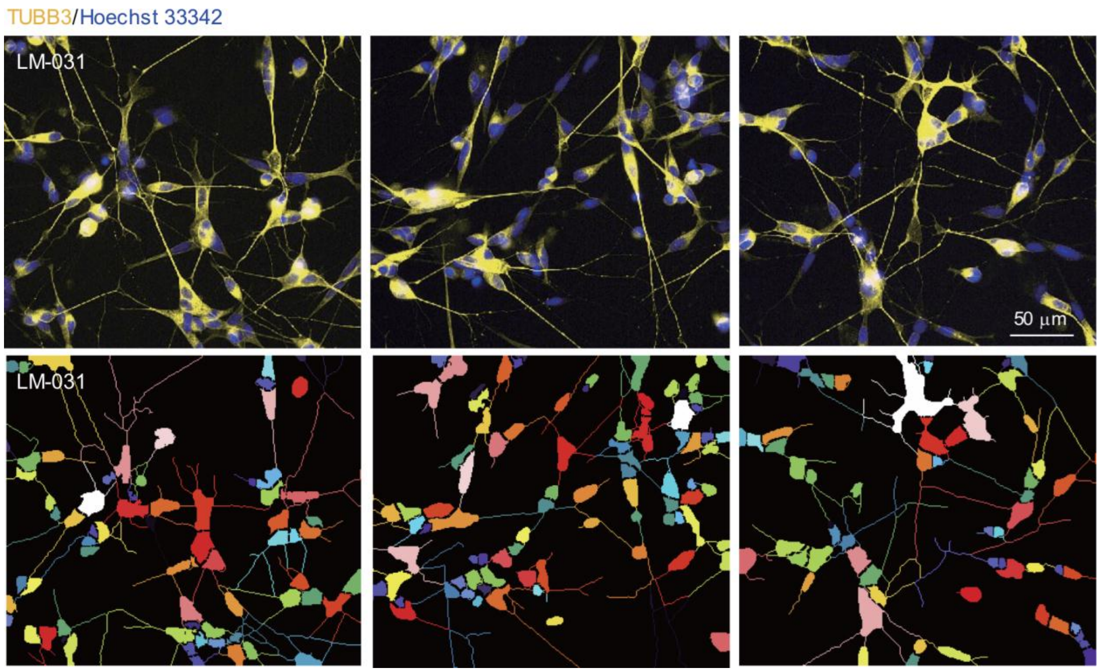

Supplementary Figure 2. Microscopic ATXN3/Q_{75}-GFP (green) or TUBB3 (yellow) images of differentiated ATXN3/Q_{75}-GFP SH-SY5Y cells untreated or treated with NC009-1, AM404, VB-037 and LM-031 (10 μM) in triplicate. In TUBB3 images, image segmentation with multi-colored mask was adopted to assign each outgrowth to a cell body for quantification.

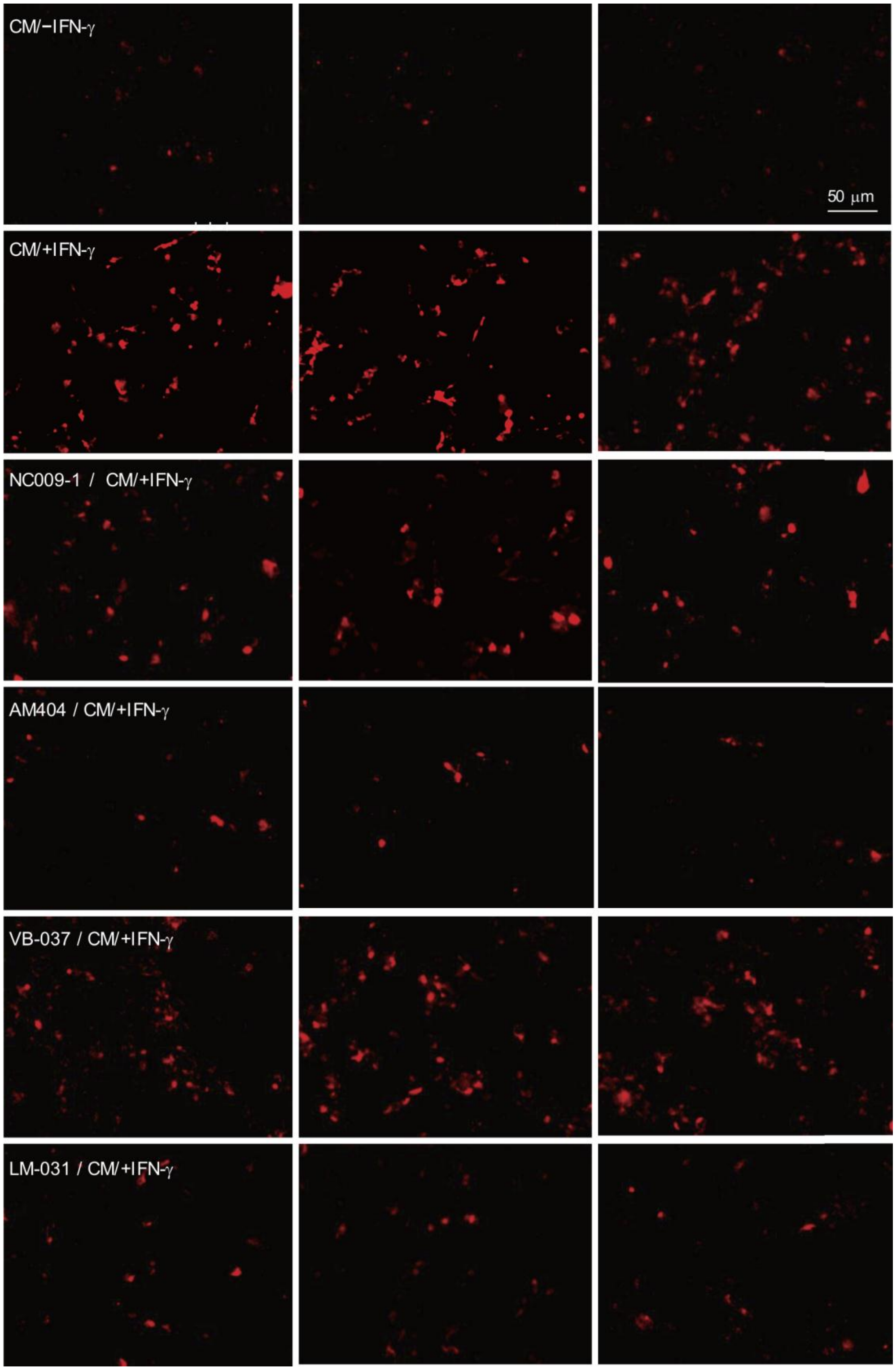

Supplementary Figure 3. Triplicated microscopic images showing the generation of ROS (red) in differentiated ATXN3/Q_{75}-GFP SH-SY5Y inflamed with HMC3 conditioned medium with or without IFN-γ stimulation, and NC009-1, AM404, VB-037 or LM-031 (10 μM)-treated cells inflamed with IFN-γ-stimulated HMC3 conditioned medium.

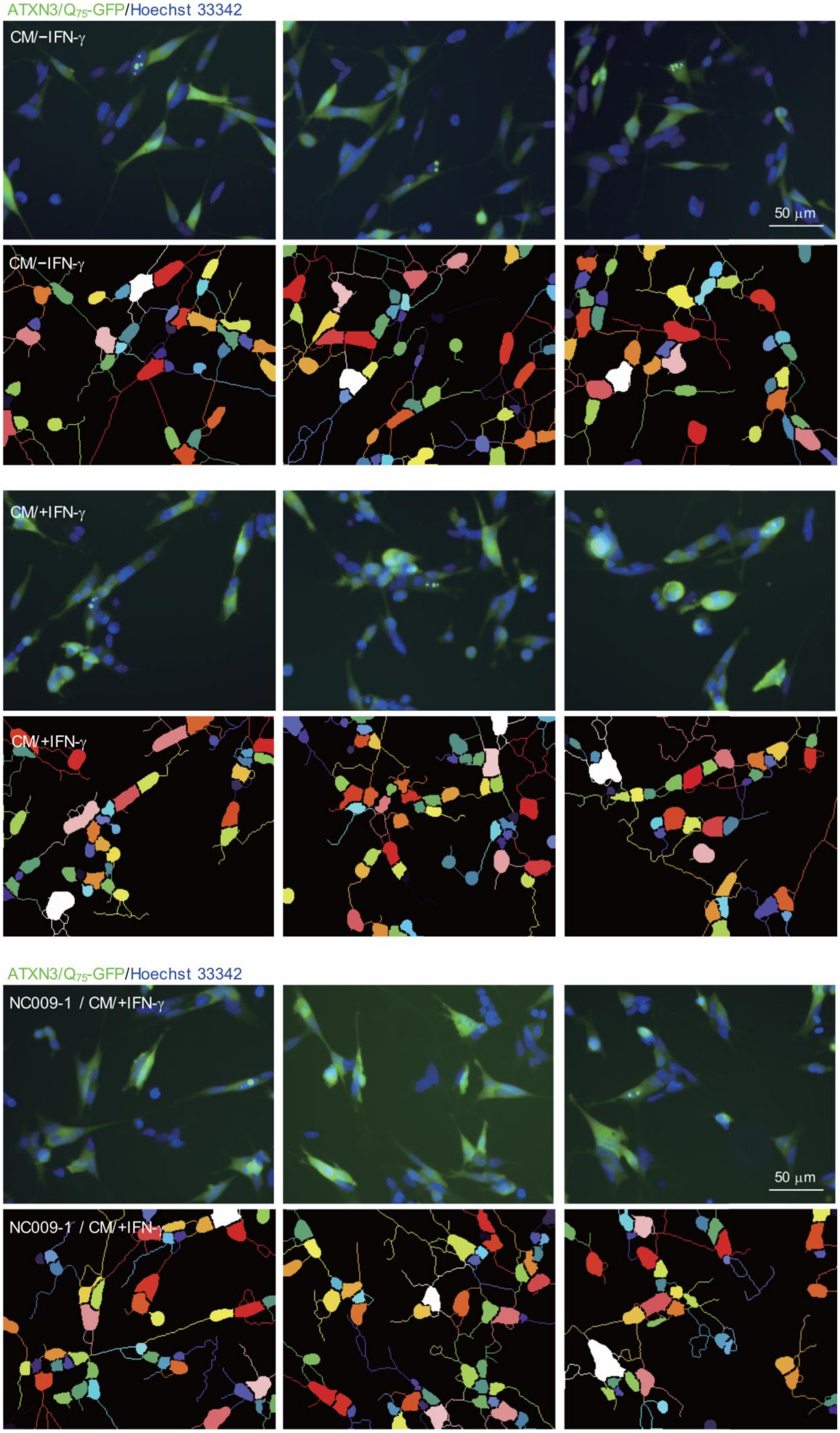
ATXN3/Q75-GFP/Hoechst 33342
CM/−IFN-γ
50 μm
CM/−IFN-γ
CM/+IFN-γ
CM/+IFN-γ
ATXN3/Q75-GFP/Hoechst 33342
NC009-1 / CM/+IFN-γ
50 μm
NC009-1 / CM/+IFN-γ

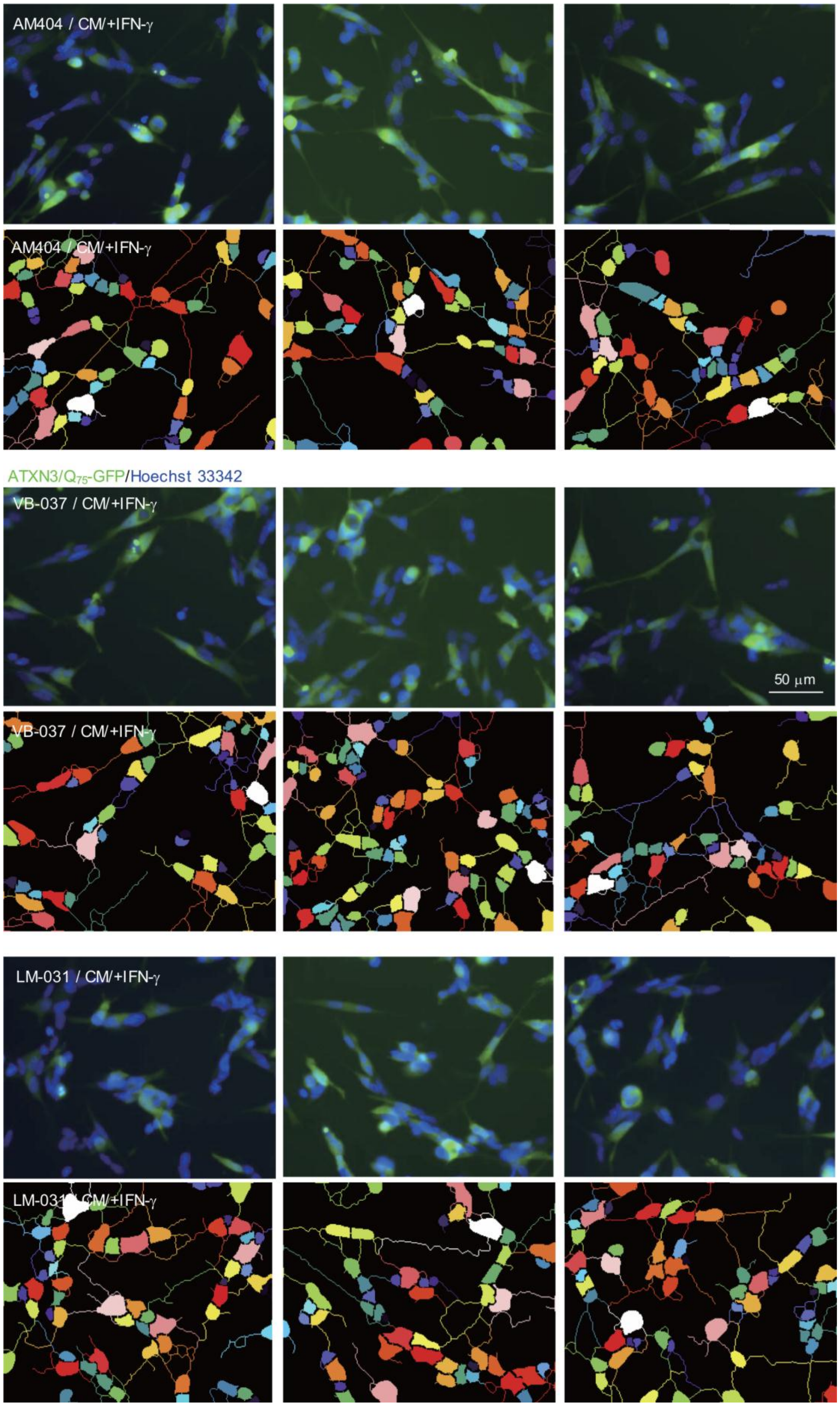

Supplementary Figure 4. Triplicated microscopic images of differentiated ATXN3/Q_{75}-GFP SH-SY5Y inflamed with HMC3 conditioned medium with or without IFN-γ stimulation, and NC009-1, AM404, VB-037 or LM-031 (10 μM)-treated cells inflamed with IFN-γ-stimulated HMC3 conditioned medium. Image segmentation with multi-colored mask was adopted to assign each outgrowth to a cell body for quantification.
